# CaFANet: Causal-Factors-Aware Attention Networks for Equipment Fault Prediction in the Internet of Things

**DOI:** 10.3390/s23167040

**Published:** 2023-08-09

**Authors:** Zhenwen Gui, Shuaishuai He, Yao Lin, Xin Nan, Xiaoyan Yin, Chase Q. Wu

**Affiliations:** 1 The 7th Rescarch Institute of Electronics Technology Group Corporation, Guangzhou 510310, China; whut_gzw@163.com; 2 School of Information Science and Technology, Northwest University, Xi’an 710127, China; heshuaishuai@stumail.nwu.edu.cn (S.H.); linyao08@stumail.nwu.edu.cn (Y.L.);; 3 Department of Data Science, New Jersey Institute of Technology, Newark, NJ 07102, USA

**Keywords:** fault prediction, industrial Internet of Things, causal factors, attention mechanism

## Abstract

Existing fault prediction algorithms based on deep learning have achieved good prediction performance. These algorithms treat all features fairly and assume that the progression of the equipment faults is stationary throughout the entire lifecycle. In fact, each feature has a different contribution to the accuracy of fault prediction, and the progress of equipment faults is non-stationary. More specifically, capturing the time point at which a fault first appears is more important for improving the accuracy of fault prediction. Moreover, the progress of the different faults of equipment varies significantly. Therefore, taking feature differences and time information into consideration, we propose a **Ca**usal-**F**actors-**A**ware Attention **Net**work, **CaFANet**, for equipment fault prediction in the Internet of Things. Experimental results and performance analysis confirm the superiority of the proposed algorithm over traditional machine learning methods with prediction accuracy improved by up to 15.3%.

## 1. Introduction

Equipment fault prediction is extremely important for the maintenance of large equipment in the Industrial Internet of Things. Compared with traditional signal-analysis-based fault diagnosis methods [1,2,3], deep-learning-based prediction algorithms [4,5,6] can achieve better performance in equipment fault prediction.

In the industrial Internet of Things, a large device is composed of multiple components, each of which has different physical characteristics during machine operation. Sensors can be deployed to collect measurements on the operation of specific components, e.g., we may deploy vibration sensors at the bearings to monitor their operation and extract features from these monitoring data, which reflect the physics of the detected component, as inputs to the deep learning framework.

However, feature extraction and interaction are calculated in a black box in a deep learning framework [7,8]. In fact, feature selection is crucial to the accuracy of equipment fault prediction. Moreover, the features extracted from the monitoring data of the equipment operational status can characterize the operational status of the equipment from different perspectives. Essentially, the representation abilities of these extracted features are different, and their contributions to equipment fault prediction are also different. Therefore, we should treat the features extracted from monitoring data accordingly when designing fault prediction algorithms.

Typically, equipment can operate normally for months or even years. Moreover, the progress of equipment faults is often non-stationary. Compared with normal operation mode, the data collected by sensors would undergo significant changes when the equipment malfunctions. What kind of data is more meaningful and how to capture these valuable data are crucial for equipment fault prediction. Therefore, we should identify the most influential parts of the monitoring data and infer the key data points closely related to equipment faults.

In this paper, we propose a **Ca**usal-**F**actors-**A**ware Attention Networks (**CaFANet**) for equipment fault prediction. Different from traditional deep learning, we first conduct causal analysis of extracted signal features to quantify the impact of each feature on the prediction accuracy, and embed the influence weight of the features on the prediction performance into the feature representation for equipment fault diagnosis. Then, we calculate local causal-factors-aware weights based on a single-layer transformer and global attention weights via a time-aware key-query attention scheme. Finally, we integrate local attention with global attention using a dynamic attention fusion strategy. Compared with state-of-the-art fault prediction algorithms, our proposed algorithm not only considers the impact of features on prediction performance but also deliberates the time information of faults in both local and global levels.

Our contributions are summarized as follows.

- We quantify the influence of features on the prediction accuracy via causal analysis and assign a causal influence weight to each feature according to its contributions to equipment fault prediction performance.
- We investigate the influence of features and time information on equipment fault prediction using a single-layer transformer, compute local weights and global weights accordingly, and finally obtain an aggregated attention weight for each sequence to achieve better equipment fault prediction performance.
- We evaluate the performance of **CaFANet** using a publicly available equipment fault prediction dataset. Compared with eleven classical baselines, the experimental results validate the effectiveness and efficiency of **CaFANet**.

The remainder of this paper is organized as follows. We survey the related works in Section 2. We present the system model and the target problem in detail in Section 3. The equipment fault prediction framework is described in Section 4. The experimental results and performance analysis are presented in Section 5. Finally, we conclude this paper in Section 6.

## 2. Related Work

Accurate equipment fault prediction can effectively reduce industrial accidents in the Industrial Internet of Things. Both traditional and modern methods can be utilized for predicting equipment faults.

The traditional methods for equipment fault diagnosis include Bayesian theory, maximum likelihood estimation, expert systems, Kalman filtering, etc. Hmida et al. [9] investigated a robust equipment fault diagnosis scheme by using a third-order Kalman filter. By taking a directed graph model into account, Jaise et al. [10] introduced a fault tree diagnosis strategy for vehicle systems. Wang et al. [11] enhanced the equipment fault signal by taking advantage of the time-frequency reduction and short-time Fourier transform and improved the performance of bearing fault diagnosis. Chen et al. [12] improved the accuracy of fault classification based on short-time Fourier transform. More importantly, this model achieved a good fault classification performance with a smaller feature scale. Shi et al. [13] designed an efficient diagnosis system by integrating an expert system and a fuzzy neural network to establish a diagnosis rule library. Zhang et al. [14] studied the stochastic resonance behavior of a coupled stochastic resonance system with time delay under the fluctuation of mass and frequency. Yan et al. [15] proposed an auxiliary indicator-based fault diagnosis system, which overcame the drawback of manually setting the modal parameters in the original singular spectral decomposition. Ding et al. [16] combined an expert system and an agent model framework to build a collaborative power grid fault diagnosis system. Although these methods based on old-fashion patterns can assist in equipment fault diagnosis, they highly rely on the professional knowledge and experience of designers. Moreover, these methods treat the extracted features equally and cannot handle the nonstationary progression of equipment faults.

There are many new technologies available for equipment fault diagnosis and prediction, such as multimodal learning, attention mechanisms, causal analysis, etc. Nan et al. [6] introduced a multimodal learning framework to combine low-quality monitoring data and high-quality monitoring data for better equipment fault prediction performance. Zhang et al. [5] realized a multidimensional neural network fusion diagnosis system with the help of a fuzzy inference system based on the data collected by multiple sensors. Zhao et al. [17] proposed a blind source extraction (BSE) method for bearing fault diagnosis based on empirical mode decomposition (EMD) and time dependence. Li et al. [18] proposed a document-level novelty detection algorithm based on attention mechanisms. Ruikun et al. [4] built a quantum neural-based network to predict fault type. Wang et al. [19] proposed an approach to discover causal structures from incomplete data by using a novel encoder and reinforcement learning. Aviles-Cruz et al. [20] designed a novel framework that used Granger-causality on a smartphone to classify and analyze human activities. In this study, to improve the performance of prediction, we design a novel equipment fault prediction framework inspired by causal analysis and an attention mechanism. We attempt to explore the intrinsic differences of features and quantify their contribution to device fault prediction performance through causal analysis. At the same time, based on an attention mechanism, we attempt to handle the non-stationary fault progression and assign different weights to serialized data points.

## 3. System Model

We consider an equipment fault prediction system mounted with *I* vibration sensors, each of which gathers *K* serialized data points about the operating status of target equipments. In longitudinal monitoring data, the monitoring data from each sensor corresponding to a specific fault is a time-ordered monitoring sequence. We obtain the time-domain characteristics of the initial sequences based on time-domain signal analysis. We extract *J* features from every data point. In summary, we have *K* serialized data points with *J* features from *I* sensors in the monitoring system.

**Figure 1:**
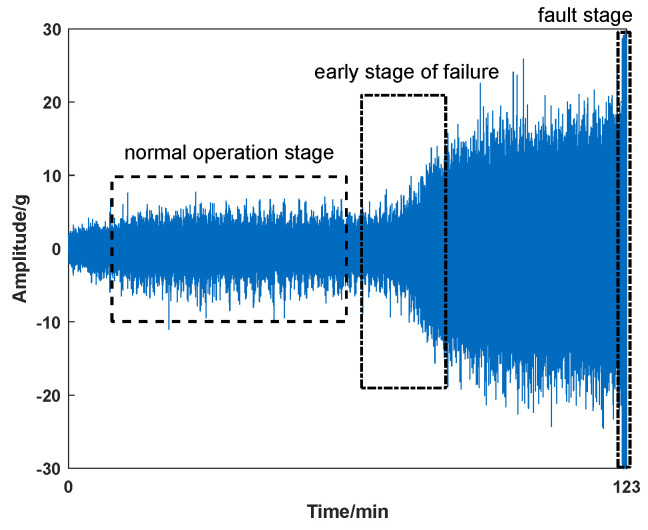
The three stages of equipment running status.

Given the collected sequences throughout the entire lifecycle, the goal of equipment fault prediction system is to predict if the target equipment would fail with one certain fault in the near future. Taking a bearing as an example, as shown in Figure 1, it has three operating states, i.e., the normal operation, the early fault, and the fault. Generally, the bearing would operate in normal mode for a long period of time. Once a fault occurs, there is a significant change in the vibration signal. However, what kind of data points are more valuable for predicting equipment faults? More importantly, how should we evaluate the contribution of these data points to the prediction performance? Due to the inherent differences between causal and non-causal features, the features that affect prediction accuracy should also be treated differently. Therefore, the objective of this study is to achieve better prediction performance by taking advantage of features and time information.

## 4. The Proposed Prediction Framework

**Figure 2:**
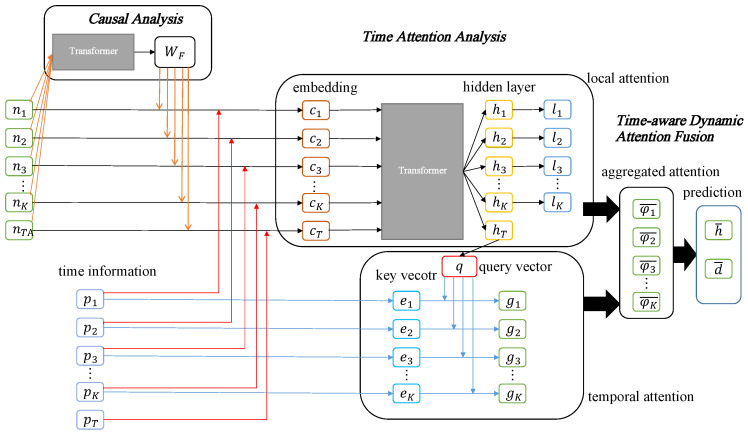
Overview of our proposed prediction framework.

Our prediction framework, **CaFANet** (**Ca**usal-**F**actors-**A**ware Attention Networks for equipment fault prediction), as shown in Figure 2, includes four components: (1) Resampling. For longitudinal monitoring data, we first preprocess the classified equipment fault samples via time-domain analysis and encode the time-domain characteristics to obtain a time-ordered monitoring sequence *N*, $N=[n_{1},n_{2},\ldots ,n_{K}]$. (2) Causal analysis. We perform causal analysis on the extracted signal features to quantify the impact of each feature on the prediction accuracy. More specifically, we evaluate the model accuracy error by removing any feature and then obtain the corresponding causal contribution weight for each feature $W_{F}$, $W_{F}=\left[W_{11},W_{12},\ldots ,W_{IJ-1},W_{IJ}\right]$. (3) Time attention analysis. We recalculate the feature embedding to obtain a new embedding sequence *C*, $C=[c_{1},c_{2},\ldots ,c_{K},c_{T}]$ by taking causal weights and the time information into account, where *C* contains the time information and causal weights, and $c_{T}$ is the feature information under failure conditions. We use transformer to obtain the hidden layer representation $H=[h_{1},h_{2},\ldots ,h_{k},h_{T}]$ of the embedded sequence *C* and then calculate the local attention score $W_{l}$. To simulate equipment faults over time, we first embed $h_{T}$ into a query vector and then embed the time difference between the occurrence of the failure and the specific data point into a key vector. We use an attention mechanism with time information to obtain the global attention score $W_{g}$ for each embedded sequence. (4) Time-aware dynamic attention fusion. We fuse the local attention score $W_{l}$ and the temporal attention score $W_{g}$ to generate the aggregated attention score ${\bar{\varphi }}_{k}$.

### 4.1. Preprocessing

Based on the equipment state monitoring information throughout the lifecycle, which includes device failure data collected by multiple deployed sensors, we extract features and predict the device operation status. The data of each device are firstly organized, classified into different fault categories, and manually annotated to form a dataset.

We resample the labeled equipment fault data and stamp each sampling point with a timestamp. The collected signal samples are organized according to the device they come from. Using the signal time-domain analysis (here, we consider the variance, root mean square difference, and mean, etc.), we obtain the time-domain characteristics of the sorted samples and make corresponding time-domain feature samples. We then standardize the extracted time-domain feature samples to generate a unified feature code as:

(1) $$s_{ijk}=\frac{f_{ijk}-Min\left(f_{ijk}\right)}{Max\left(f_{ijk}\right)-Min\left(f_{ijk}\right)}\times \gamma \times \left(i\times J\right)\times j,$$

where $s_{ijk}$ is the feature code of the *k*th serialized point of the *j*th feature gathered by sensor *i*, i=1,2,…,I, j=1,2,…,J, k=1,2,…,K, $Max(f_{ijk})$ and $Min(f_{ijk})$ are the corresponding maximum and minimum values, respectively, and γ is a coefficient related to the size of the feature space. Note that *I* is the total number of deployed sensors, *J* is the number of time domain features extracted from monitoring data, and *K* is the total number of monitoring sample sequences for each device.

We discretize the continuous values of feature codes obtained by Equation (1), each feature code $s_{ijk}$ can be converted into a corresponding $b_{ijk}$ in a binary form represented by ${\left\{0,1\right\}}^{o}$, o=γ×I×J, and all $s_{ijk}$ are rounded down. Let *N* denote the sequence of samples, and we have

(2) $$N=[n_{1},n_{2},\cdots ,n_{k},\cdots ,n_{K}],$$

where $n_{k}=\left[b_{11k},b_{12k},\cdots ,b_{IJk}\right]$, and $n_{k}$ is the *k*-th sequence.

### 4.2. Causal Analysis

Traditional fault prediction methods for large-scale equipment treat all features equally [6]. In fact, each feature actually makes different contribution to the prediction accuracy because of its unique representation ability. More specifically, the performance of prediction depends on the selection of features. Therefore, we use causal analysis to quantify the influence of characteristics on prediction performance. The function to quantify the contribution of each feature to the accuracy of equipment fault prediction is expressed as follows:

(3) $$\Delta_{\varepsilon ,f_{ij}}=\varepsilon_{F\setminus \left\{f_{ij}\right\}}-\varepsilon_{F},$$

where $\Delta_{\varepsilon ,f_{ij}}$ is the effect of feature $f_{ij}$ on fault prediction, $\varepsilon_{F}$ is the error in fault prediction, and $\varepsilon_{F\setminus \left\{f_{ij}\right\}}$ is the error in fault prediction without feature $f_{ij}$. We use *M* to denote the embedding sequence of the model and $M\setminus \left\{f_{ij}\right\}$ to denote the embedding sequence without feature $f_{ij}$. $M=[m_{1},m_{2},\cdots ,m_{k}]$, $M\setminus \left\{f_{ij}\right\}=[m_{1}^{*},m_{2}^{*},$ and $\cdots ,m_{K}^{*}]$, $m_{k}$ can be calculated as follows:

(4) $$m_{k}=W_{m}n_{k}+b_{m},$$

where $m_{k}$ is the embedding data of the *k*th sequence of the embedded sequence *M*, $n_{k}$ is the binary input of the feature code $f_{ijk}$ of the *k*th sequence, $W_{m}$ is the initialization weight matrix, $W_{m}\in R^{v}$, $b_{m}$ is the bias vector, $b_{m}\in R^{v}$, and *v* is the dimension of $w_{m}$ and $b_{m}$.

The traditional transformer-based schemes are mainly applied to solve natural language processing problems but are rarely used in the Internet of Things. There are two advantages to applying transformer to the fault prediction of large equipment. Firstly, the transformer allows the vector to use the self-attention mechanism every time to learn its relationship with other samples from other sensors deployed in the same equipment. Secondly, the structure of the transformer provides the ability to calculate multiple samples in parallel. Thus, we use an attention-mechanism-based single-layer transformer as a model for causal analysis, denoted by TF(∗), and the label of the prediction result denoted by $\hat{e}$, and we have:

(5) $${\hat{e}}_{\varepsilon_{F}}=TF\left(M\right),$$

(6) $${\hat{e}}_{\varepsilon_{F\setminus \left\{f_{ij}\right\}}}=TF\left(M\setminus \left\{f_{ij}\right\}\right),$$

where ${\hat{e}}_{\varepsilon_{F}}$ and ${\hat{e}}_{\varepsilon_{F\setminus \left\{f_{ij}\right\}}}$ denote the model prediction labels with and without feature $f_{ij}$, respectively.

Let *e* denote the true label and the cross-entropy loss function L denote the predicted error. The fault prediction error of Equation (3) can be expressed as:

(7) $$\varepsilon_{F}=L\left(e,{\hat{e}}_{\varepsilon_{F}}\right),$$

(8) $$\varepsilon_{F\setminus \left\{f_{ij}\right\}}=L\left(e,{\hat{e}}_{\varepsilon_{F\setminus \left\{f_{ij}\right\}}}\right).$$

We can measure the contribution of each feature to the prediction accuracy via Equations (3)–(8). Then, each feature is assigned a weight according to its influence on prediction performance as follows:

(9) $$W_{f_{ij}}=\frac{\Delta_{\varepsilon ,f_{ij}}}{|\sum \Delta_{\varepsilon ,f_{ij}}|}.$$

Then, we obtain the causal weight $W_{F}=\left[W_{f_{11}},W_{f_{12}},\cdots ,W_{f_{ij}}\right]$.

### 4.3. Time Attention Analysis

We recalculate the feature code using the causal influence weights obtained in Section 4.2 as follows:

(10) $$s_{ijk}^{W}=W_{f_{ij}}\frac{f_{ijk}-\min f_{ijk}}{\max f_{ijk}-\min f_{ijk}}\times \gamma \times \left(i\times J\right)\times j,$$

where $s_{ijk}^{W}$ is the updated feature code of the *k*-th serialized point of the *j*-th feature from sensor *i*, i=1,2,…,I, j=1,2,…,J, k=1,2,…,K.

Similarly, we can obtain the sample sequence $N^{W}=\left[N_{1}^{W},N_{2}^{W},\cdots ,N_{K-1}^{W},N_{K}^{W},N_{T}^{W}\right]$ that combines the causal influence weights, where $N_{K}^{W}$ is the binary input of the updated feature code $s_{ijk}^{W}$, and $N_{T}^{W}$ is the feature representation of the specific fault of the equipment in the failure state. Note that the $N_{T}^{W}$ is the same for all samples when predicting a particular type of fault.

The moment when the operating state of the equipment transitions from normal to abnormal is crucial. To improve the performance of equipment fault prediction, it is necessary to capture this moment. The closer the sampling point is to the fault point, the more attention it deserves and the greater the weight it should be given. Therefore, we should take time information into account. More specifically, we embed time information and then use the updated vectors also as inputs. Firstly, since the time information and the feature vector are not in the same latent space, to characterize the importance of features associated with the time information, we need to embed the time information into the feature vector space as follows:

(11) $$z_{k}=W_{z}\left(1-tanh\left({\left(\frac{{}_{2p_{k}}}{T}\right)}^{2}\right)\right)+b_{z},$$

where $z_{k}$ is the embedded time vector, tanh is the hyperbolic tangent function, *T* is the sampling time of the last serialized point in the sequence, $p_{k}$ is the time difference between the current sampling point and the last sampling point, $p_{k}=T-t*k$, $W_{z}$ and $b_{z}$ are the initialization weight matrix and bias vector, respectively, $w^{z}\in R^{v}$, and $b^{z}\in R^{v}$.

The sequence can be generated by embedding with time information and causal influence weights as follows:

(12) $$c_{k}=z_{k}+\left(W_{c}N_{k}^{W}+b_{c}\right),$$

where $c_{k}$ is the embedded conterpart of the *k*th sequence. The sequence $C=[c_{1},c_{2},\cdots ,c_{K},c_{T}]$ can be obtained by Equation (12). Note that the $c_{T}$ is the same for all samples.

We use a single-layer transformer (denoted by TF()) to learn the relationship between the embedding sequences and the equipment faults as follows:

(13) $$\left[h_{1},h_{2},\cdots ,h_{K},h_{T}\right]=TF\left(\left[c_{1},c_{2},\cdots ,c_{K},c_{T}\right]\right),$$

where $h_{k}$ is the hidden representation of $c_{k}$ via the transformer, k=1,2,⋯,K, and $h_{T}$ is the representation of $c_{T}$, which is the embedding sequence of a specific device fault.

Due to non-stationary fault progression, we should review all sample points, rather than only focusing on the current sample point, when we analyze equipment faults and identify the turning point of the equipment’s operating status, i.e., the key point when it transitions from the normal operating state to the abnormal state. To carry out this fault diagnosis, we calculate a local attention score for each sample point as follows:

(14) $$u_{k}=W_{u}^{⊤}h_{k}+b_{u},$$

where $W_{u}$ and $b_{u}$ are the weight matrix and bias vector, respectively, $W^{u}\in R^{l}$, and $b_{u}\in R^{}$. We use a softmax layer to generate local attention weights based on the local attention score $U=[u_{1},u_{2},\cdots ,u_{K}]$. We have

(15) $$W_{l}=Softmax\left(\left[u_{1},u_{2},\cdots ,u_{K}\right]\right)=\left[l_{1},l_{2},\cdots ,l_{K}\right].$$

We learn a local attention weight, which reflects the importance of sample points on the accuracy of equipment fault prediction, for each sample via the aforementioned time-aware transformer. Not only does a single sample point need to be focused on, but attention to the progress of the equipment operation status is more important for equipment fault prediction. Compared to existing samples, if the gathered data show only slight changes over a long period of time, it is not necessary to collect and analyze the data at regular intervals. Only when there are significant fluctuations in the monitoring data do more samples need to be collected, which helps to predict equipment failures more accurately. Therefore, the time interval between samples is crucial for fault prediction. We introduce a key-query attention model to simulate the process. Firstly, we convert the hidden representation $h_{T}$ obtained by Equation (13) into a query vector *q* as follows:

(16) $$x=ReLU(W_{x}h_{T}+b_{x}),$$

where $W_{x}\in R^{l\times q}$ and $b_{x}\in R^{q}$ are the weights and bias matrix, respectively, *l* is the dimension of $h_{k}$, *q* is the dimension of $b_{x}$, and ReLU is an activation function that only maintains positive values.

To capture the importance of the time information itself during the equipment operation process, we use the time difference $p_{k}$ between the fault point and the sample as the key vector $e_{k}$ for the attention mechanism as follows:

(17) $$e_{k}=tanh\left(W_{e}\left(1-tanh\left({\left(\frac{{}_{2p_{k}}}{T}\right)}^{2}\right)\right)+b_{e}\right),$$

where $W_{e}\in R^{l\times q}$ and $b_{e}\in R^{q}$ are parameters to be learned, $e_{k}$ is the key vector of the *k*th sequence, *l* is the dimension of $h_{k}$, *q* is the dimension of $b_{e}$, and $E=[e_{1},e_{2},\cdots ,e_{K}]$ is the set of key vectors.

Combined with the query vector *x* derived from Equation (16), we can obtain the global attention score $r_{k}$ as follows:

(18) $$r_{k}=\frac{x⊤e_{k}}{\sqrt[{}]{r}}.$$

We can obtain global attention weights $W_{g}$ based on the global attention score $r_{k}$ via a softmax layer as follows:

(19) $$W_{g}=Softmax\left(\left[r_{1},r_{2},\cdots ,r_{K}\right]\right)=\left[g_{1},g_{2},\cdots ,g_{K}\right],$$

where $g_{K}$ is the global time-aware attention weight of the *K*th sequence.

### 4.4. Attention Fusion Strategy

The two attention vectors obtained earlier pay attention to different perspectives for equipment fault prediction. $W_{l}$ focuses on the characteristics of each embedded sequence, while $W_{g}$ concentrates on the time information of all samples. To obtain more accurate prediction results, we should take feature and time information into account. Therefore, we introduce a dynamic attention fusion strategy that integrates local attention with global attention. More specifically, we embed $h_{T}$ into a new space and normalize it as follow:

(20) $$v=Softmax\left(W_{v}h_{T}+b_{v}\right)=\left[a_{l},a_{g}\right],$$

where $W_{v}\in R^{2\times l}$, $b_{v}\in R^{2}$, *l* is the dimension of $h_{K}$. We then obtain an aggregated attention weight for each sequence based on local weights and global weights as follows:

(21) $$\varphi_{k}=l_{k}*a_{l}+g_{k}*a_{g}.$$

Finally, we can obtain the final attention score $\gamma^{\prime }$ by normalizing the aggregated attention weights for each vector as follows:

(22) $$\gamma^{\prime }=\frac{\varphi_{k}}{\sum_{k}\varphi_{k}}.$$

The final attention score combines the influence of the features and time information on the device fault prediction. The feature analysis focuses on the signal values of the dataset throughout the entire lifecycle, while the time information analysis pays attention to the changes in the time interval between samples over a long period. Integrating signal features and time information can better capture the contribution of features to the prediction results.

We obtain the representation of a sample based on the hidden layer representation obtained by Equation (13) and the final attention score obtained by Equation (22) as follows:

(23) $$h^{\prime }=\sum \gamma^{\prime }h_{k}.$$

We perform a binary prediction of equipment faults based on the the representation obtained by Equation (23) via a softmax layer as follows:

(24) $$d^{\prime }=Softmax\left(W_{d}h^{\prime }+b_{d}\right),$$

where $W_{d}$ and $b_{d}$ are parameters to be learned, $W_{d}\in R^{2\times l}$, $b_{d}\in R^{2}$.

We define a loss function to learn all parameters, let θ denote all the parameters and *d* denote the ground truth, and use the cross entropy between the predicted probabilities $d^{\prime }$ and the actual value *d* as the loss function as follows:

(25) $$L\left(\theta \right)=-\frac{1}{\left|N\right|}\sum_{n=1}^{\left|N\right|}\left(d^{⊤}log\left(d^{\prime }\right)+{\left(1-d\right)}^{⊤}log\left(1-d^{\prime }\right)\right),$$

where |N| is the total number of samples.

## 5. Experimental Evaluation

In this section, we describe the experiments we conducted and compare the performance of the proposed prediction model and baselines on a publicly available equipment failure prediction dataset.

### 5.1. Dataset and Data Preprocessing

We performed experiments on the XJTU-SY bearing dataset [21], which measures the operating conditions of bearings and is widely applied to the fault prediction and remaining life estimation of the bearings. The data are collected by vibration sensors deployed on the adjustable speed generator shaft, supporting the transmission bearings, the hydraulic drive load control system, and the testing transmission bearings, etc. The monitored data can be used to evaluate if the equipment has malfunctioned.

This dataset, XJTU-SY, contains information on five bearings, each with three operating modes, and a total of 15 sets of samples throughout the entire lifecycle, each of which serves as a sub-dataset. More specifically, the first bearing has three sub-datasets, denoted as Bearing 1_1, Bearing 1_2 and Bearing 1_3. Meanwhile, we set the sampling frequency as 25.6 kHz, the average sampling interval as 1 min, and each sampling time as 1.28 s. Therefore, we obtained 32,769 signals from each sensor per minute.

**Figure 3:**
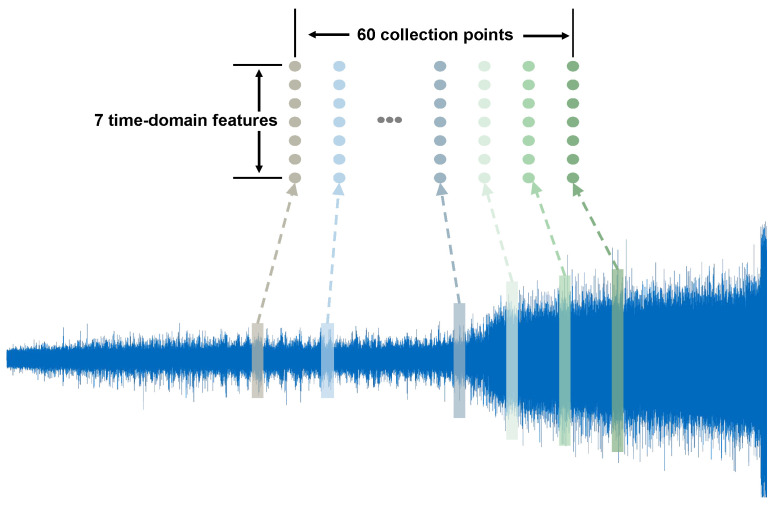
The resampling of the signal sequences.

Given the limited number of operating modes in the XJTU-SY bearing dataset, we enhanced the dataset by resampling because of the periodicity of the original samples. The information about each operating mode of each bearing can be divided into a normal operation stage, an early fault stage, and a fault stage. Due to the limited value of the equipment fault data for fault prediction, signals at the fault stage should not be considered. At the same time, the information entropy contained in the signal during the normal operation stage is very small; so, the information at this stage should be considered as little as possible. On the other hand, the signals corresponding to the period near the occurrence of the fault are extremely important for fault prediction and require more attention. Taking the samples at a speed of 2100 r/min and a radial force of 12 kN as an example, starting from the time when the equipment is turned on, every 2000 signals were treated as one data point and then labeled. The characteristics of these data points were obtained through numerical analysis, and thus the sequential points of each operating mode for each bearing during the whole life cycle were obtained. We sampled 20 random data points during the normal operation stage and 40 data points during the early fault stage to create a sequence, as shown in Figure 3. One thousand sequences for each operating mode of each bearing were generated as a dataset, 60% of which were used as the training set, 20% as the validation set, and 20% as the testing set.

Obviously, each sub-dataset contained the vibration signal of a bearing under one certain operating mode throughout the entire lifecycle. To verify the validity of the proposed model, we merged the sub-datasets belonging to different fault types to obtain four combined datasets. We created Dataset 1 by merging the sub-datasets Bearing 2_1 and Bearing 2_3, with inner race wear as the prediction target. We created Dataset 2 by merging the sub-datasets Bearing 2_3 and Bearing 2_5, with cage fracture as the prediction target. We created Dataset 3 by merging the sub-datasets Bearing 1_1, Bearing 2_3, and Bearing 2_5 datasets, with outer race wear as the prediction target. We created Dataset 4 by merging all the sub-datasets, with outer race fracture as the prediction target.

### 5.2. Time-Domain Signal Features

We selected seven time-domain features for equipment fault prediction and conduct causal analysis to measure the influence of these features on the model. The features are described as follows:

- **Variance (VAR)**. The VAR is used to measure the statistical dispersion of the signal. The larger the variance, the greater the signal variation. The smaller the variance, the smaller the signal fluctuation.
- **Root Mean Square (RMS)**. The RMS is not sensitive to early vibration signals but has good stability.
- **Average Value (AV)**. The AV can be used to measure the stability of signals and reflect the static properties of signal fluctuations.
- **Kurtosis (KU)**. KU can be used to measure the probability distribution of random variables. KU has good performance for faults with pulse signals. However, KU fails and have poor stability when a fault occurs.
- **Skewness (SK)**. SK can be used to measure the degree and direction of data distribution deviation and can characterize the degree of numerical asymmetry distribution. SK has good performance in the early fault stage but fails after a fault occurs.
- **Crest Factor (CF)**. The CF is defined as the ratio of the peak to the rectified average value and can be used to judge whether there are pulses in the signal.
- **Margin Factor (MF)**. The MF is defined as the ratio of the signal peak to the root square amplitude and is more sensitive to changes in the signal.

### 5.3. Experiments Settings

All our experiments were based on PyTorch, implemented on the Windows 10 operating system, run on 32 GB of RAM and GeForce RTX 3070 Ti GPU. The batch size was set as 50, the learning rate was set as 0.01, the space coefficient γ was set as 1400, the dropout was set as 0.5, the dimension of the hidden space *I* for prediction was 256, and Adam [22] was chosen as the optimizer. We rnm each prediction algorithm 10 times on each dataset and obtained the average of these 10 results as the final result.

### 5.4. Metrics

To evaluate the effectiveness and efficiency of the prediction model, we used accuracy (*Acc*), precision (*Pre*), and recall (Recall) as metrics as follows:

(26) $$Acc=\sum_{i=0}^{N}\frac{P_{i,i}}{\sum_{i=0,j=0}^{N}P_{i,j}},$$

(27) $$Pre=\sum_{i=0}^{N}\frac{P_{i,i}}{\sum_{j=0}^{N}P_{j,i}},$$

(28) $$Recall=\sum_{i=0}^{N}\frac{P_{i,i}}{\sum_{j=0}^{N}P_{i,j}},$$

where *N* is the total number of samples, and $P_{i,j}$ indicates that the predicted label is *i* and the actual label is *j*. More specifically, the prediction is correct only when *i* equals *j*.

### 5.5. Baselines

To demonstrate the superiority of the proposed prediction algorithm, we chose three types of algorithms as baselines.

- The first category of baselines was classical machine learning algorithms, including **SVM** (Support Vector Machine) [23], **LR** (Linear Regression) [24] and **RF** (Random Forest) [25].
- The second category of baselines was RNN-based algorithms, including **LSTM** (Long Short-Term Memory) [26], **GRU** (Gated Recurrent Unit) [27], and **DA-RNN** (Dual-stage Attention-based Recurrent Neural Network) [28]. These algorithms are the basic framework of most prediction algorithms.
- The third category of baselines was algorithms that have achieved good performance in bearing fault prediction in recent years. **CNN** (Convolutional Neural Network) [29] is the classical and effective classification algorithm. **DFC-CNN** (Deep Fully Convolutional Neural Network) [30] is based on CNN and spectrogram transform for prediction. **CNN-LSTM** (multiscale CNN and LSTM) [31] can learn the original signal and encode it directly. **GRU-HA** (Gate Recurrent Unit and Hybrid Autoencoder) [32] can automatically learn the features of sequences. **DA-AE** (Deep Wavelet Autoencoder) [33] is an unsupervised learning algorithm and uses the original vibration signal for training.

### 5.6. Performance Analysis

**Prediction Accuracy Comparison**. As shown in Table 1, compared with the 11 benchmark algorithms, our proposed algorithm achieved the highest fault prediction accuracy. Compared with LR, our algorithm had an accuracy improvement of 15.3% on Dataset 4. Our algorithm assigned different weights to features that had different contributions to the prediction results and considered the impact of the time information on fault prediction. Therefore, our algorithm achieved optimal prediction performance on Dataset 4.

**Prediction Performance Comparison**. A comparison of 12 algorithms on Dataset 2, as shown in Table 2. Our method outperformed all the benchmarks on dataset 2 in terms of accuracy, precision, and recall. Compared with LR, our algorithm improved the accuracy by 7%, the accuracy ratio by 8%, and the recall rate by 6% on Dataset 2.

**Effect of the Time-domain Features**. Table 3 lists the impact of each time-domain feature on the accuracy of fault prediction under different fault types. CaFANet explores features that have the higher correlation with the predicted target. As expected, **KU**, **CF** and **MF** were more sensitive to the early numerical changes in bearing faults, thus having a greater impact on fault prediction tasks.

**Figure 4:**
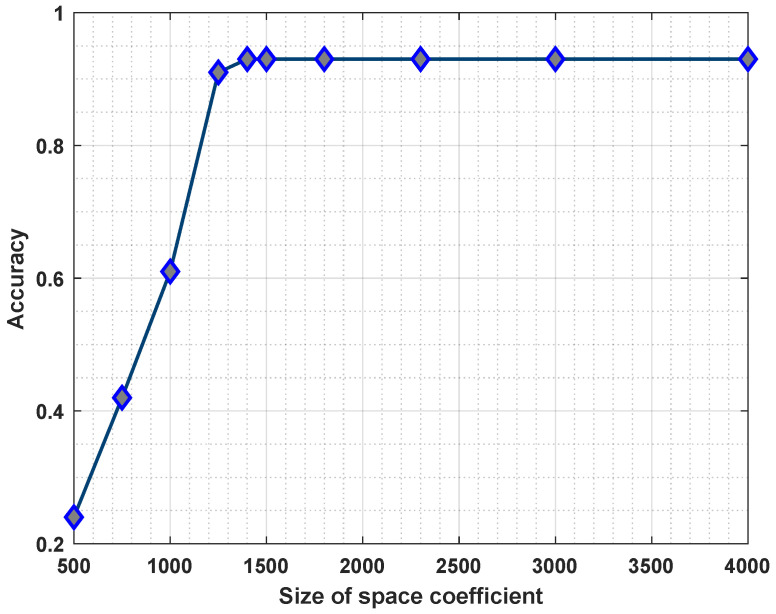
The effect of the space coefficient on accuracy.

**Figure 5:**
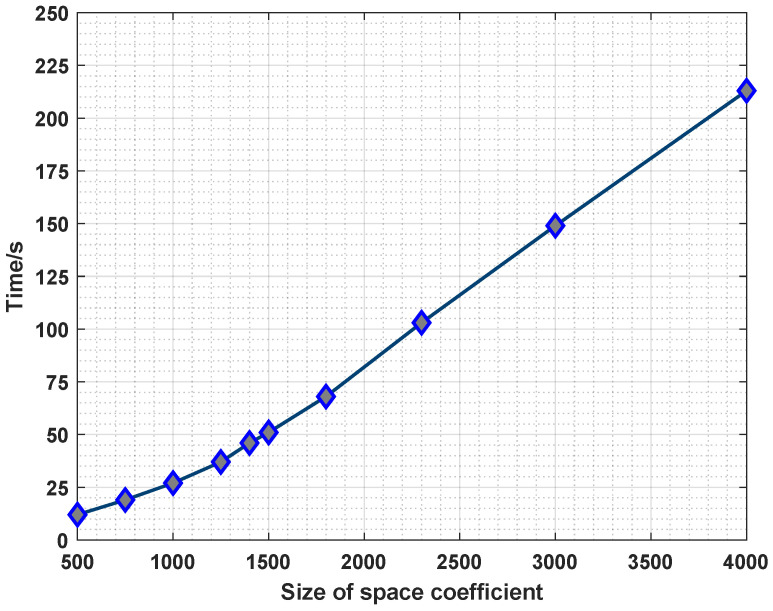
Running time comparison of the different space coefficients.

**Effect of the Space Coefficients**. The results shown in Figure 4 confirmed the impact of the space coefficient sizes on the fault prediction accuracy, which increased with the increase in the space coefficients. The larger the space coefficients, the higher the complexity of the encoding process and the higher the accuracy of the fault prediction. The running time increases linearly accordingly, as shown in Figure 5. However, if we chose smaller spatial coefficients, it is difficult to effectively express the relationship between features, resulting in low fault prediction accuracy.

**Figure 6:**
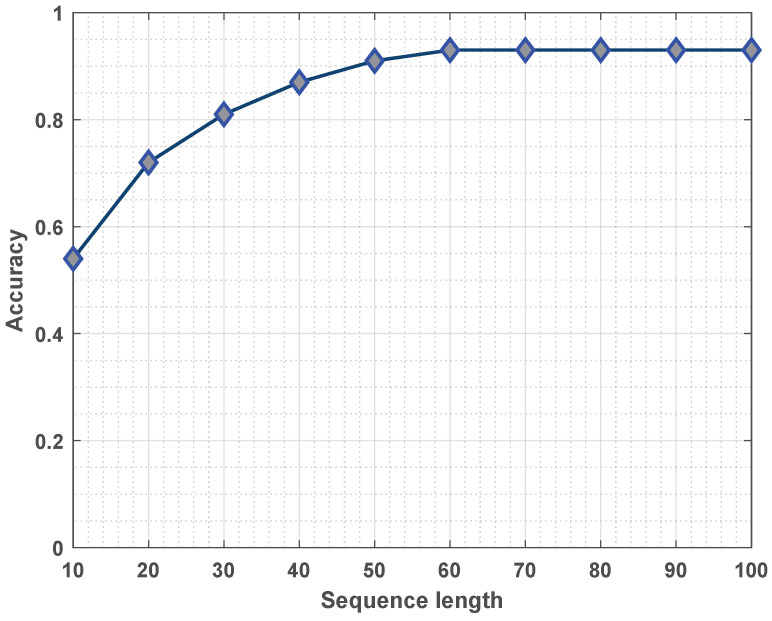
The effect of the sequence length on the accuracy.

**Effect of the Sequence Length**. Figure 6 illustrates the impact of the sequence length on the accuracy of fault prediction. Overall, the prediction accuracy increased with the increase in the sequence length. Actually, the impact of the sequence length on the prediction accuracy depended on the number of data points in the early fault stage.

**Ablation Study**. An ablation study on the impact of different components of the prediction model on accuracy was conducted by removing the causal analysis, global time attention, and time information embedding, respectively. As shown in Table 4, the prediction accuracy variation denoted the gap between the performance with and without the specific component. From Table 4, we observe that time embedding had the strongest impact on the prediction accuracy.

## 6. Conclusions

In this paper, we designed a causal-factors-aware attention network for predicting the fault types of bearings in the Internet of Things. Firstly, we measured the contribution of the time-domain features to the prediction model using causal analysis. Focusing on time-domain features with larger contributions, we then introduced a local attention mechanism to assign weights to the embedding sequence. After embedding time information, the features of the key stages were amplified, and the transformer with time-aware information was used to find the data points closely related to the faults. Finally, we reassigned weights to each signal sequence to predict the equipment faults effectively and efficiently. In this paper, we confirmed the importance of time information to prediction accuracy. Moreover, the proposed algorithm can provide guidance on the feature selection of equipment failure prediction in the industrial Internet of Things. Good prediction performance depends on high-quality data. However, it is usually difficult to obtain the monitoring data of the large equipment due to its confidential nature in the industrial Internet of Things. Even though device monitoring data is available, obtaining device fault information is still very challenging. Provided with samples and corresponding monitoring sequences, CaFaNet is able to perform interpretable equipment fault prediction and has potentials for practical use in production settings.

## Figures and Tables

**Table 1:** Accuracy comparison of Dataset 4.

Methods	Dataset 1	Dataset 2	Dataset 3	Dataset 4
SVM	0.901	0.893	0.861	0.855
RF	0.883	0.876	0.856	0.847
LR	0.896	0.847	0.768	0.749
LSTM	0.887	0.910	0.858	0.839
GRU	0.870	0.902	0.847	0.826
CNN	0.885	0.882	0.887	0.829
DFC-CNN	0.897	0.896	0.892	0.852
DA-RNN	0.892	0.885	0.889	0.876
CNN-LSTM	0.899	0.894	0.899	0.868
GRU-HA	0.916	0.911	0.900	0.892
DW-AE	0.922	0.915	0.904	0.896
CaFANet	**0.930**	**0.924**	**0.913**	**0.902**

**Table 2:** Performance comparison on Dataset 2.

Methods	Acc	Pre	Reacll
SVM	0.893	0.884	0.899
RF	0.876	0.895	0.864
LR	0.847	0.861	0.839
LSTM	0.910	0.924	0.898
GRU	0.902	0.935	0.877
CNN	0.882	0.901	0.869
DFC-CNN	0.896	0.906	0.892
DA-RNN	0.885	0.896	0.872
CNN-LSTM	0.894	0.899	0.886
GRU-HA	0.911	0.934	0.890
DW-AE	0.915	0.941	0.895
CaFANet	**0.924**	**0.949**	**0.905**

**Table 3:** Causal analysis comparison of the time-domain features.

Feature	Dataset 1	Dataset 2	Dataset 3	Dataset 4	Overall
Var	0.0413	0.0398	0.0421	0.0402	0.0409
RMS	0.0279	0.0283	0.0265	0.0288	0.0279
AV	0.0379	0.0369	0.0357	0.0370	0.0369
KU	0.0957	0.0968	0.0912	0.0899	0.0934
SK	0.0284	0.0237	0.0256	0.0274	0.0263
CF	0.0715	0.0768	0.0742	0.0739	0.0741
MF	0.0734	0.0796	0.0722	0.0785	0.0759

**Table 4:** Influence of components of the prediction model on accuracy.

Models	Acc
No causal analysis	0.883
No global time attention	0.866
No embedding time information	0.806
Full model	**0.924**

## Data Availability

The public dataset, XJTU-SY bearing datasets, can be downloaded at the following link: https://share.weiyun.com/5zLCgBL (accessed on 1 January 2021).

## Abbreviations

In this study, we used the following abbreviations:

CaFANet	Causal-Factor-Aware Attention Networks for Equipment Fault Prediction
SVM	Support Vector Machine
LR	Linear Regression
RF	Random Forest
LSTM	Long Short-Term Memory
GRU	Gated Recurrent Unit
DA-RNN	Dual-stage Attention-based Recurrent Neural Network
CNN	Convolutional Neural Network
DEF-CNN	Deep Fully Convolutional Neural Network
CNN-LSTM	Multiscale CNN and LSTM
GRU-HA	Gate Recurrent Unit and Hybrid Autoencoder
DA-AE	Deep Wavelet Autoencoder
